# Muscle Recovery and Nutrition

**DOI:** 10.3390/nu14122416

**Published:** 2022-06-10

**Authors:** Alberto Caballero-García, Alfredo Córdova-Martínez

**Affiliations:** 1 Department of Anatomy and Radiology, Faculty of Health Sciences, GIR Physical Exercise and Aging, Campus Los Pajaritos, University of Valladolid, 42004 Soria, Spain; 2 Department of Biochemistry, Molecular Biology and Physiology, Faculty of Health Sciences, GIR Physical Exercise and Aging, Campus Duques de Soria, University of Valladolid, 42004 Soria, Spain

## Introduction

Recovery strategies, both in the general population and in athletes, must be aimed at the main causes of fatigue. Any physical activity involves an increase in metabolism and the functionality of the functional systems of our body [1,2]. There are many studies on proteins, amino acids, immediate principles and metabolic regulators (vitamins and minerals) that have demonstrated their importance and effectiveness in muscle recovery [1,2,3].

During intense exercise, the muscles develop fatigue and weakness throughout a limited period of time. The process is exacerbated in the case of subjects that do not exercise regularly, as well as during very intense exercise bouts. In these particular cases, muscle damage may take days to recover [4]. To carry out a recovery process through nutritional intervention can be key, both in the general population and in athletes in particular. A faster and more efficient recovery will allow athletes to train harder and respond to training more positively, for which an adequate nutritional intake is essential.

Throughout this special issue on “Muscle recovery and nutrition”, different aspects have been addressed according to the objectives set.

In a systematic review carried by Drobnic et al. [2] the possible use of Coenzyme Q10 Supplementation on the recovery of athletes was analysed. The conclusion of this manuscript was that the use of Coenzyme Q10 seems to offer a good profile in the control of an oxidative pattern with a certain anti-inflammatory activity at the cellular level in response to exercise. It can therefore be seen as a protective and recuperative substance rather than an ergogenic substance in itself.

Drobnic et al. [5], in an observational study (over 12 weeks), analysed the effect of krill oil on HS-Omega-3 index levels and plasma choline on the performance and recovery after intense exercise. They focused the study on the recovery of HS-Omega-3 index levels, post-exercise plasma choline recovery and free radical scavenging after high-power physical training. The authors observed that the combined administration of n-3 PUFAs, choline and astaxanthin in the form of krill oil did not demonstrate a clear improvement in athlete performance. However, the authors indicate that the optimisation of the HS-Omega-3 index before training, as well as choline and oxidative stress after training, may be key in optimising athlete performance and recovery.

In one study we analyzed the use of citrulline as a supplement to modulate sarcopenia syndrome [4]. Sarcopenia is a process associated with aging that starts from the age of 30 years and progresses, resulting in strength reduction due to a decrease in muscle mass [6]. Muscle wasting and degeneration is a consequence of muscle atrophy and muscle cell death, leading to the loss of strength and muscle mass [7,8,9]. In this study [4], a group of healthy people over 60 years of age were supplemented with citrulline and performed a physical activity protocol suitable for their age range. Taking into account the characteristics of our study, the results obtained show a modest potency but are very important from a recovery point of view. Nevertheless, in line with this research we also found studies which demonstrate a significant benefit in the use of amino acids such as citrulline [10,11]. However, the beneficial effects of citrulline in combination with exercise in the management of sarcopenia require further research.

Paratthakonkun et al. [12] presented a study about the effects of crocodile blood (CB) supplementation on the inflammatory, biochemical and functional performance responses to muscle-damaging exercise and to evaluate the short-term safety of CB in healthy males. The Thailand food and drug administration (FDA) has approved that crocodile blood (CB) products shall not exceed the maximum allowance level of 1 g per day as a dietary supplementation for human consumption [13]. The authors have concluded that an 18-day supplementation of 1 g day^−^^1^ of CB helps to maintain peak muscle force or DOMS compared to a placebo after eccentric exercise. In addition, biochemical analysis provided evidence that 1 g day^−^^1^ of CB supplementation should be safe for human consumption.

In another study [14], the effects of the combination of whey protein and casein in the ratio of 80:20 (“whey protein: casein” or “casein:whey protein”) were investigated as a basis for the proportions of fast absorption and slow absorption proteins in the ratios of human breast milk (HBM), which is a gold standard for protein in quality and nutrient contents [15]. Such effects could provide changes in the amino acid concentrations of branched-chain amino acids in plasma and cause changes in the bioavailability at the peak period of permanence of branched-chain amino acids in the blood circulation, the final metabolites of the protein metabolism and cause delayed onset muscle soreness (DOMS) after a single bout of fasting or a resistance exercise session. The authors have concluded that, despite no positive effects being observed in the protein metabolism markers, the amino acid kinetics highlighted that the whey protein (WP) seems to be the most effective supplement to increase the leucine concentration in plasma, and there was no advantage in the association of WP and casein (CAS) in the amino acids peak period of permanence when compared to WP itself; however, it minimized muscle soreness compared to CAS and placebo.

Glutamine, as well as other amino acids, favours a muscle anabolic state, increasing protein synthesis [16,17]. Street et al. [18] indicated that glutamine supplementation could attenuate the inflammatory response after eccentric exercise. Legault et al. [17] observed that glutamine supplementation reduces muscle soreness, indicating a possible correlation with less muscle damage. However, at the moment, there are no conclusive studies about the use of glutamine supplementation in athletes for immunomodulation and/or anabolic processes. Moreover, there is even less evidence regarding the role of glutamine in preventing exercise-induced muscle damage. In our study [4], we have analysed the effect of glutamine supplementation on recovery after eccentric muscle damage in professional basketball players. In conclusion, the data presented show that glutamine supplementation results in a decrease in circulating muscle damage markers, accompanied by an adequate balance between the response of the catabolic and anabolic hormones and the stability of the leucocyte cell numbers. We hypothesize that the control of these specific parameters could help to prevent the inflammation and stress provoked by highly strenuous exercise.

Mieszkowski et al. [19] studied the anti-inflammatory effects of vitamin D during exercise training. Previously, Choi et al. [20] communicated that inflammation induced by high-intensity exercise is significantly reduced upon vitamin D supplementation in an animal model. The authors studied a group of 35 semi-professional ultramarathon runners (males) that participated in the Lower Silesian Mountain Runs Festival 2018 Ultra Marathon Race. In the study, Mieszkowski et al. [19] showed that the administration of a single high dose of vitamin D significantly blunts the rise of proinflammatory cytokine levels after an ultramarathon, even though serum levels of 25(OH)D are significantly elevated after the run. These observations imply that the ultramarathon-induced increase in 25(OH)D levels is not sufficiently high so as to reduce inflammation. Hence, improving the vitamin D status before an endurance competition might be a good alternative to the use of anti-inflammatory drugs that are so often relied on in sports.

In a meta-analysis about the effects of Vitamin D in post-exercise muscle recovery, we observed [21] that although vitamin D seems to be effective against the muscular inflammatory process, the role in post-exercise recovery by modulating the release of muscle biomarkers remains to be demonstrated [22]. We suggest that following investigations include cytokine determinations, and the use of a longer administration time or higher doses of supplementation would be variables to take into account. From a practical point of view, vitamin D supplementation serves to normalize and optimize its own blood levels. However, additional studies with comparable protocols are necessary to reach more solid evidences regarding post-exercise muscle recovery.

As has been communicated by several authors, athletes have a high risk of vitamin D deficiency. Despite the evident benefit of vitamin D in muscle function, particularly in recovery from inflammation caused by exercise, there are still few experimental studies that demonstrate an improvement in performance after vitamin D supplementation. The musculoskeletal benefits occur when deficient or insufficient circulating levels of vitamin D (20–30 ng/mL) are corrected by providing supplements. However, no improvements in muscle function and performance are observed when subjects with already normal circulating levels of vitamin D (50 ng/mL) are supplemented. However, there is still controversy regarding the optimal levels for supplementation. Daily vitamin D requirements have been estimated between 3000 and 5000 IU (75–125 g/day) to meet essential needs for all tissues and cells in the body [23]. However, the intakes recommended by experts might not only cover daily metabolic requirements but might also favour the storage of vitamin D and increase its availability. In our opinion, a supplementation with vitamin D requires, first, monitoring the long-term circulating levels of vitamin D. In addition, according to our experience with sports professionals, the daily doses recommended by the European Endocrine Society [24] maintained in the long term could mimic the effects reached by a megadose supplementation in the short term.
